# Comparison of Mechanical and Barrier Properties of Al_2_O_3_/TiO_2_/ZrO_2_ Layers in Oxide–Hydroxyapatite Sandwich Composite Coatings Deposited by Sol–Gel Method on Ti6Al7Nb Alloy

**DOI:** 10.3390/ma13030502

**Published:** 2020-01-21

**Authors:** Bożena Pietrzyk, Daniel Kucharski, Łukasz Kołodziejczyk, Sebastian Miszczak, Mateusz Fijalkowski

**Affiliations:** 1 Institute of Materials Science and Engineering, Faculty of Mechanical Engineering, Lodz University of Technology, Stefanowskiego Str. 1/15, 90-924 Lodz, Poland; 2 Institute for Nanomaterials, Advanced Technologies and Innovation, Technical University of Liberec, Studentská 1402/2, 460 01 Liberec 1, Czech Republic

**Keywords:** hydroxyapatite, oxide, layered, composite, coating, sol–gel, barrier, permeation

## Abstract

In this study, coatings of different oxides (TiO_2_, Al_2_O_3_, ZrO_2_) and hydroxyapatite (HAp) as well as sandwich composite hydroxyapatite with an oxides sublayer (oxide+HAp) were deposited on Ti6Al7Nb alloy using the sol–gel dip-coating method. The coatings were characterized in terms of morphology (optical microscope), surface topography (AFM), thickness (ellipsometry), and crystal structure (XRD/GIXRD). The mechanical properties of the coatings—hardness, Young’s modulus, and adhesion to the substrate—were examined using nanoindentation and scratch tests. The barrier properties of the coatings against the migration of aluminum ions were examined by measuring their concentration after soaking in Hank’s balanced salt solution (HBSS) with the use of optical emission spectrometry of inductively coupled plasma (ICPOES). It was found that all the oxide and HAp coatings reduced the permeation of Al ions from the Ti6Al7Nb alloy substrate. The best features revealed an Al_2_O_3_ layer that had excellent barrier properties and the best adhesion to the substrate. Al_2_O_3_ as a sublayer significantly improved the properties of the sandwich composite HAp coating.

## 1. Introduction

In modern medicine, advance is closely related not only to new drugs and treatment methods but also to the development of materials engineered to interact with biological systems. Such materials, called biomaterials, are used primarily in restorative medicine, where they replace the tissues of a living organism as the implants [1]. A modern trend in the engineering of implantable biomaterials is the modification of their surfaces by producing coatings [2,3,4]. These coatings not only enable the functioning of implants in the living organism’s environment, but also promote integration with its tissue [2,5]. One of the most important materials widely used for this purpose is hydroxyapatite (HAp)–Ca_10_(PO_4_)_6_(OH)_2_ [6,7]. In addition to improving biocompatibility, HAp exhibits high bioactivity and contributes to intensified osseointegration [8,9]. Hydroxyapatite owes such properties to its chemical and biological similarity to bones, promoting the creation of new bone tissue [9]. Unfortunately, the attractiveness of hydroxyapatite as a bioactive material is limited by its relatively poor adhesion to implant substrates [10], which is closely related to the method of its deposition.

There are numerous methods of producing HAp coatings, such as plasma spraying [11,12], laser deposition [13,14], sputtering [15,16] high-speed spray coating [17,18], electrodeposition [19,20], or the sol–gel method [21,22]. Most of them are characterized by sudden temperature changes, phase transitions, and/or rapid kinetic deposition on coated surfaces. This often leads to the formation of disordered, porous, amorphous–crystalline hydroxyapatite structures, with a high degree of defect (i.e., cracks), low cohesion, and mediocre adhesion—due to mismatching of the physicochemical properties of coatings compared to metallic substrates [23,24]. There is also a tendency to dissolve HAp coatings due to their biochemical interaction with the living tissue, which can lead to the exposure of the implant’s metallic substrate [10].

The simultaneous achievement of appropriate bioactive and mechanical properties of hydroxyapatite on a metal substrate can be achieved by applying a composite coating using an HAp and oxide mixture. Numerous studies [25,26,27,28,29] have investigated the variation of the microstructural, compositional, morphological, or surface properties of such coatings and their influence on the bioactivity, mechanical, and bonding strength. There is also another approach, consisting of the production of layered composite coatings, where the oxide layer, e.g., TiO_2_ [30,31,32], ZrO_2_ [32,33,34], Al_2_O_3_ [26,31], and SiO_2_ [30], is applied directly to the substrate and the HAp layer is deposited on top of it. This solution seems to have some advantages, e.g., better control over the composition and morphology of individual layers, as well as potentially much better isolation of the substrate in the event of dissolution of the HAp layer after implantation. It means that after HAp layer dissolution, the permeation of ions from metallic implants can be limited by an insoluble oxide layer.

In this study, coatings of different oxides (TiO_2_, Al_2_O_3_, and ZrO_2_) and hydroxyapatite (HAp) as well as sandwich composite hydroxyapatite with an oxides sublayer (oxide+HAp) were deposited on Ti6Al7Nb. The Ti6Al7Nb is a second-generation alpha–beta titanium alloy with higher corrosion resistance and biotolerance [35] compared to a commonly used Ti6Al4V alloy.

The coatings were made using a sol–gel dip-coating method—a simple and convenient technique for producing ceramic materials, including biomaterials [36,37]. The sol–gel method is fast and effective [38], it has a low process temperature, and can be used to producing oxide [39] and hydroxyapatite [40] coatings with good control of their properties.

Obtained coatings were characterized in terms of morphology and topography, phase structure, mechanical properties, adhesion, and barrier properties in HBSS (Hank’s balanced salt solution).

The novelty of this work is a comprehensive approach to the role of the oxide layer in limiting the permeation of Al ions from the substrate while ensuring the proper mechanical properties of the entire oxide–hydroxyapatite coating. Combined with the use of a consistent and inexpensive sol–gel technique for the deposition of all layers, this will allow the production of coatings that restrict the potentially negative effects of the Al ions’ interaction with human tissue during and after the osseointegration process.

## 2. Materials and Methods

### 2.1. Coatings Preparation

A sol for the hydroxyapatite coating was prepared by mixing of 1 mole solutions of Ca(NO_3_)_2_·4H_2_O and (C_2_H_5_O)_3_PO in ethanol. In the prepared sol, the ratio of calcium to phosphorus was Ca/P = 1. After mixing, the sol was aged in a sealed vessel for 16 h at 60 °C.

Titania (TiO_2_) sol was prepared by dissolving titanium (IV) butoxide (Ti[O(CH_2_)_3_CH_3_]_4_, Sigma-Aldrich, Stainheim, Germany) in ethanol, adding water and acetic acid. The molar ratios of the components were [Ti]:[H_2_O]:[CH_3_COOH] = 1:10:1.

Alumina (Al_2_O_3_) sol was made on the base of aluminum-tri-sec-butoxide (Al[OCH(CH_3_)C_2_H_5_]_3_, Sigma-Aldrich, Stainheim, Germany) dissolved in ethanol with the addition of acetic acid CH_3_COOH as a catalyst. The molar ratios of the components were [Al]:[H_2_O]:[CH_3_COOH] = 1:2:2.

Zirconia (ZrO_2_) sol was prepared by dissolving a zirconium (IV) isopropoxide precursor (Zr[O(CH_2_)_2_CH_3_)]_4_, Sigma-Aldrich) in 1-propanol with the addition of acetylacetone (AcAc) (C_5_H_8_O_2_, Sigma-Aldrich, Stainheim, Germany) and water with a molar ratio of [Zr]:[AcAc]:[H_2_O] = 1:2:2.

Substrates for deposition of the coatings were Ti6Al7Nb alloy and monocrystalline Si (100) wafers. Ti6Al7Nb alloy substrates were prepared by cutting a 15 mm diameter rod into 5 mm thick slices, grinding, and polishing. Before the deposition of coatings, both Ti6Al7Nb alloy and Si substrates were cleaned in an ultrasonic bath in ethanol.

**Figure 1:**
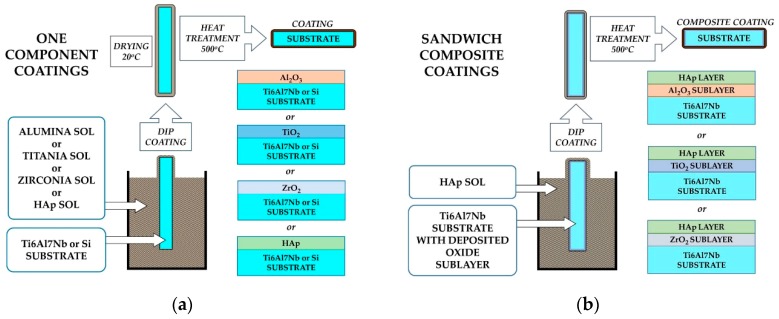
Manufacturing scheme of one component (**a**) and sandwich composite (**b**) coatings.

On such prepared substrates, oxides coatings (TiO_2_, Al_2_O_3_, or ZrO_2_) were deposited by the sol–gel method, using a dip-coating technique and a TL 0.01 Dip Coater (MTI Corporation, Richmond, CA, USA). The schema of manufacturing of coatings is presented in Figure 1. Ti6Al7Nb alloy has been used as substrate for all kinds of investigated coatings. Silicon has been the substrate only for the one-component coatings predicted for the thickness measurements.

For all the oxide coatings, samples were pulled out from relevant sol with a withdrawing speed of 1.2 mm/s, dried for 15 min at ambient condition, and then annealed at 500 °C for 15 min. The whole procedure was repeated twice for TiO_2_ and Al_2_O_3_, and three times for ZrO_2_ to obtain oxide coatings of similar thickness. For samples intended for testing, the barrier properties’ ZrO_2_ deposition procedure was repeated twice (this coating was signed as TZrO_2_). The hydroxyapatite (HAp) coatings were deposited in the same way, but after withdrawing from sol, they were rapidly heated (without drying) at the temperature of 500 °C to avoid dewetting [41]. The HAp layer was deposited both on bare substrate and onto previously obtained oxide layers to prepare sandwich composite oxide–hydroxyapatite coatings. Parameters of the oxide and HAp coating processes are depicted in the Table 1.

### 2.2. Characterization of Coatings

The morphology and topography of the obtained coatings was characterized using an optical microscope, a Nikon Eclipse MA100 with a digital camera (Nikon Corporation, Tokyo, Japan) and a Multimode 5 atomic force microscope (AFM, Bruker Corporation, Santa Barbara, CA, USA) operating in tapping mode. All investigations were performed under ambient conditions. For each sample, the area of 5 × 5 µm was scanned. Commercial silicon cantilevers type HQ:NSC15 (MicroMasch, Tallin, Estonia) with a nominal tip radius of approximately 8 nm, nominal cantilever spring constant of 40 N/m, and nominal resonance frequency of 325 Hz were used. AFM image acquisition was performed with the use of Nanoscope 7.3 software, and further image processing was done using Nanoscope Analysis 1.5 (Bruker Corporation) and MountainsMap 5 (Digital Surf, Besançon, France) software.

The thickness of the oxide layers of coatings was measured on the silicon substrate with the help of a VASE spectroscopic ellipsometer (J.A. Woollam Co., Inc., Lincoln, NE, USA) using the EMA and Cauchy model. The thickness of the HAp layer was measured using an SEM microscope Jeol JSM-6610 (JEOL Ltd., Tokyo, JAPAN) on the cross-section of HAp coating on a silicon substrate.

The crystalline structure of the Al_2_O_3_ and TiO_2_ coatings was analyzed with a D-500 X-ray diffractometer (XRD) (Siemens, Munich, Germany) using Co-K*α* characteristic radiation and a graphite monochromator. The crystalline structure of the ZrO_2_ and HAp coatings was determined based on diffraction patterns obtained using a Panalytical Empyrean GIXRD diffractometer (Empyrean, Malvern Panalytical, Worcestershire, UK) and Cu-K*α* (for ZrO_2_) as well as Co-K*α* (for HAp) characteristic radiation. The GIXRD scan was collected with a grazing incidence angle of 1 degree. The phase composition was identified using X’Pert Pro computer software, with support of the ICDD database.

The mechanical properties of the coatings were measured using the nanoindentation technique on the Nano Indenter G200 system (KLA Corporation, Milpitas CA, USA). For nanoindentation, a diamond Berkovich tip (Micro Star Technologies, Huntsville, AL, USA) and the continuous stiffness measurement (CSM) mode were used, allowing the computation of the hardness continuously during the indentation loading. The tip shape was calibrated by conducting experiments on a fused silica standard. The tests have been carried out at a strain rate of 0.05 s^−1^. The harmonic displacement was 2 nm and the frequency was 45 Hz. The data were analyzed using the approach of Oliver and Pharr [42], and nine experiments were performed on each sample.

Scratch tests were performed on a Bruker UMT-2 (Bruker Corporation, Billerica, MA, USA) micromechanical materials tester, with a linear applied load of 0–20 N with a scratching rate of 10 mm/min. Scratches of 1 mm length were performed using Rockwell tip with a radius of 200 µm. The delamination force L_c_ was determined based on the photo analysis of the scratch.

The investigations of barrier properties were performed by Al ions concentration measurements in incubation fluid. Coated Ti6Al7Nb alloy samples as well as the uncoated substrates were placed in polypropylene bottles in 50 mL of Hank’s balanced salt solution (HBSS) with the following ionic composition ([mol/dm^3^]): Na^+^ 1.42 × 10^−1^; K^+^ 5.81 × 10^−3^; Mg^2+^ 8.11 × 10^−4^; Ca^2+^ 1.26 × 10^−3^; Cl^−^ 1.45 × 10^−1^; HPO_4_^2−^ 7.78 × 10^−4^; SO_4_^2−^ 8.11 × 10^−4^; and CO_3_^2−^ 4.17 × 10^−1^ (Biological Industries, Israel). The solution was adjusted to pH 4.0 with drops of 1% HCl. Before immersing in the solution, all the samples were subjected to a sterilization process consisting of washing in anhydrous ethanol and drying for 10 min at 350 °C. The sealed sample bottles as well as the clear solution (without the sample) were kept in an incubator at 37 °C for 9 weeks. Then, the concentration of aluminum ions in each solution was determined using inductively coupled plasma optical emission spectrometer (ICPOES) model Optima 2100DV (Perkin Elmer, Waltham, MA, USA). Each measurement was performed twice using 15 mL of solution without any dilution or mineralization of the sample. The mass *M_Al_* of the aluminum ions in particular test solutions after the test was calculated from the following formula

M_Al_ = Cp × V (1)

where:

*M_Al_*—mass of Al ions in the solution [µg];
*Cp*—measured ion concentration [µg/dm^3^];
*V*—volume of the liquid in which the samples were submerged [dm^3^].

The permeability of the coatings was determined as the mass of the ions released from the sample per surface unit and calculated from the following formula

Pp = (M_Al_ – M_0_)/Sp (2)

where:

Pp—mass of Al ions released per surface unit [µg/cm^2^];
M_Al_—mass of Al ions in the solution [µg];
M_0_—mass of Al ions in the relative solution (HBSS without sample) [µg];
Sp—sample surface in contact with the liquid [cm^2^].

The concentration of Al ions in relative solution (without sample) was measured as 0.060 µg/dm^3^.

## 3. Results and Discussion

### 3.1. Morphology, Roughness, and Thickness of Coatings

**Figure 2:**
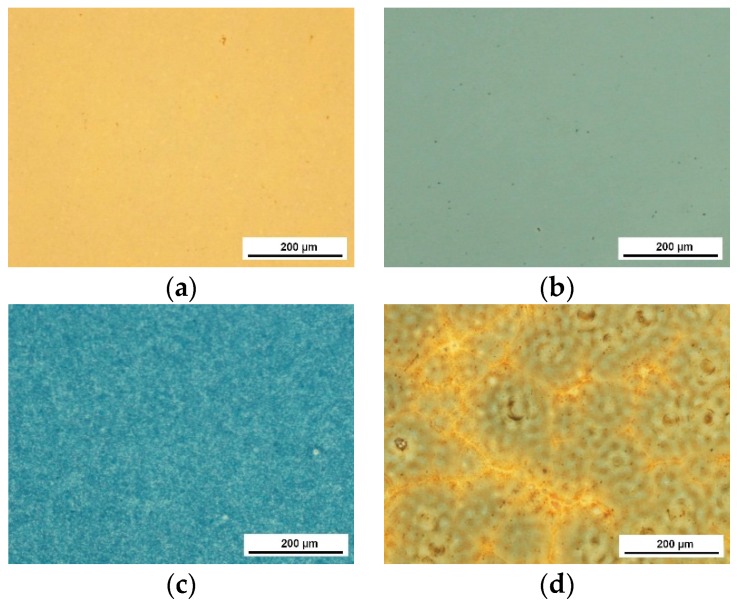
Microscopic images of one-component coatings: TiO_2_ (**a**), Al_2_O_3_ (**b**), ZrO_2_ (**c**), and HAp (**d**) deposited on Ti6Al7Nb alloy substrates.

**Figure 3:**
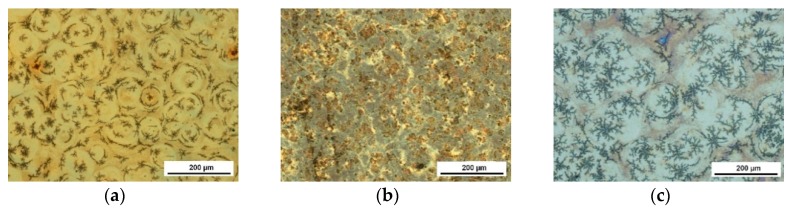
Microscopic images of composite coatings: TiO_2_+HAp (**a**), Al_2_O_3_+HAp (**b**), and ZrO_2_+HAp (**c**) deposited on Ti6Al7Nb alloy substrates.

As a result of the dip-coating deposition process, one-component coatings as well as sandwich composite coatings made of oxides and hydroxyapatite have been obtained. Microscopic images of one-component coatings deposited on Ti6Al7Nb are presented in Figure 2 and images of sandwich composite coatings are presented in Figure 3.

All examined coatings were of good quality without cracks, delamination, or other discontinuities. The one-component oxide coatings were smooth and homogeneous. The morphology of HAp (Figure 2d) and sandwich composite coatings (Figure 3) results from the properties of the HAp sol. The phenomenon of dewetting observed for this sol can lead to instability of the liquid layer after deposition and its division into individual drops. The choice of specific coating process parameters and the use of rapid heat treatment prevents this phenomenon, but one can notice the formation of small differences in the thickness of the coating in microareas, which lead to different forms of its crystallization.

**Figure 4:**
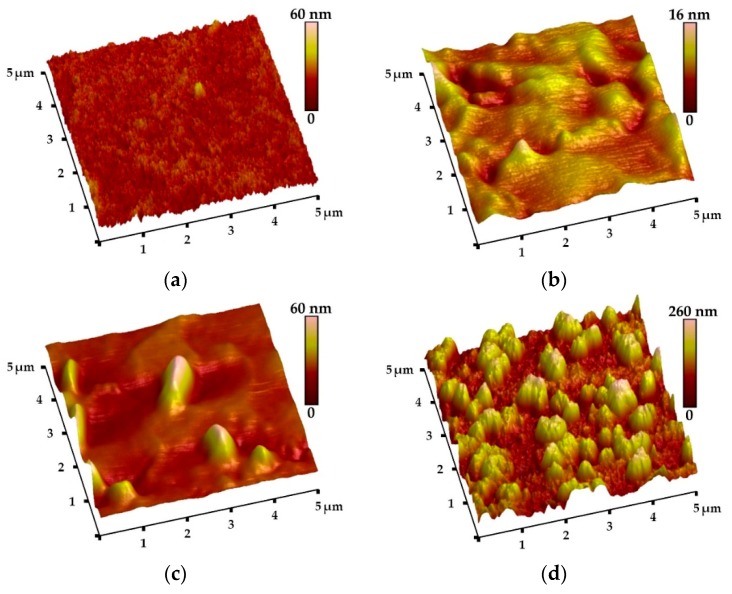
Atomic force microscope (AFM) topography of one-component coatings: TiO_2_ (**a**), Al_2_O_3_ (**b**), ZrO_2_ (**c**), and HAp (**d**), deposited on Ti6Al7Nb alloy substrates.

The complementing of the morphology of coatings is the study of their surface topography. The AFM images of one-component coatings are shown in Figure 4. They confirm that the oxide coatings are smooth, with few particles on the surface, while the HAp coating has a much more developed surface. The high roughness of the HAp surface promotes its bioactivity and active bonding with living bone [2,3,8,9], so it is beneficial due to the role of this layer in the coating.

The roughness parameters of all analyzed coatings, obtained from AFM topography analysis, are presented in Table 2. At first glance, a clear difference in both Ra and Rz parameters between synthesized coatings can be observed. For TiO_2_ and ZrO_2_ sublayers, the average values of both parameters are rather low (Ra of 2.27 ± 0.34 nm and 1.45 ± 1.34 nm, Rz of 15.3 ± 2.96 nm and 8.69 ± 7.74 nm, respectively), but they are also a few times higher than those for the bare substrate and Al_2_O_3_ coating (Ra of 0.34 ± 0.10 nm and 0.60 ± 0.27 nm, Rz of 2.37 ± 0.66 nm and 2.85 ± 1.47 nm, respectively). The highest roughness parameters were registered for the HAp coating (Ra = 29.1 ± 7.82 nm, Rz = 131 ± 23.2 nm). One can notice high values of the standard deviations for the last-mentioned sample, which can be explained by the nonhomogeneous porous surface geometry.

The influence of the topography of coatings was also visible in the results of the thickness measurements presented in Table 3. The value of the standard deviation of the HAp coating thickness is much higher than for oxide coatings. As in the case of roughness, this is due to the developed complex structure of the surface of the HAp coating. The thickness of the HAp layer (175 ± 25 nm) was at least twice as high as the thickness of the oxide layers (49–86 nm). The thickness of oxides depended on type of sol and number of repetition of the deposition process. The topography, roughness, and thickness of the individual layers were advantageous from the point of view of the role envisaged for these layers in the composite coating.

### 3.2. Phase Composition

**Figure 5:**
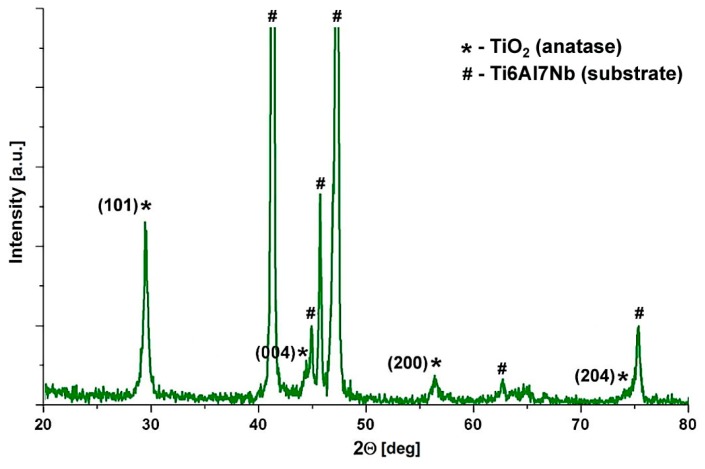
Diffraction pattern of TiO_2_ coating deposited on a Ti6Al7Nb alloy substrate.

The phase composition of one-component coatings was determined on the basis of diffraction patterns obtained using X-ray diffractometry (XRD/GIXRD). Figure 5 shows a diffractogram of the TiO_2_ coating deposited on a Ti6Al7Nb alloy substrate.

In addition to strong reflections coming from the substrate (identified using ICDD card No. 04-002-5207), reflections from the crystalline phases of the coating are also visible. Based on ICDD card No. 04-009-8171, they were assigned to planes (101), (004), (200), and (204) of the anatase unit cell—one of the TiO_2_ crystalline polymorphs typically obtained in this thermal treatment temperature range [43].

**Figure 6:**
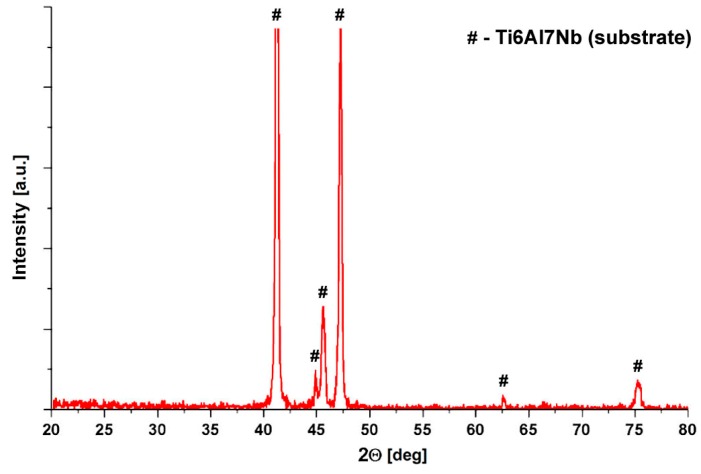
Diffraction pattern of Al_2_O_3_ coating deposited on Ti6Al7Nb alloy substrate.

The XRD spectra of an Al_2_O_3_ coating on Ti6Al7Nb alloy can be seen in Figure 6.

The diffraction pattern of Al_2_O_3_ coating lacks identifiable reflections from the coating. The absence of alumina reflections indicates that the coating is amorphous. This is due to the low heat treatment temperature, which is insufficient to initiate the crystallization of the Al_2_O_3_ matrix [44]. Previous work has shown that aluminum oxide obtained in the described sol–gel process consists of amorphous alumina, which has been transformed from aluminum hydroxide oxide AlO(OH) and can crystallize into *γ*-Al_2_O_3_ after annealing at temperature not lower than 500 °C [45].

**Figure 7:**
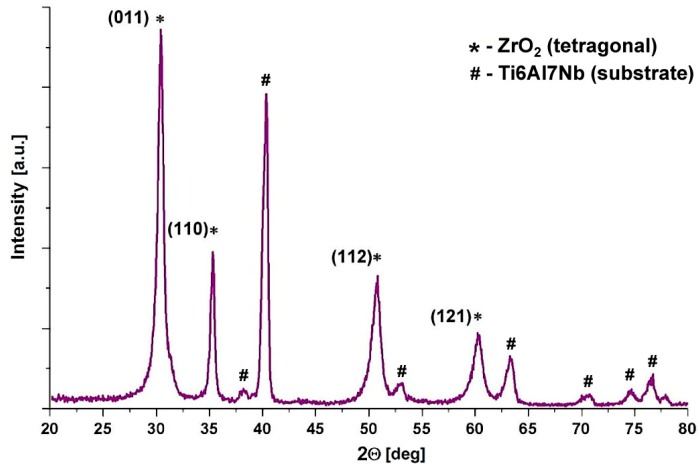
Diffraction pattern of ZrO_2_ coating deposited on the Ti6Al7Nb alloy substrate.

The diffraction pattern of the ZrO_2_ coating on Ti6Al7Nb alloy is shown in Figure 7.

The reflections marked in the spectrum (2Θ: 30.27°, 35.25°, 50.38°, and 60.2°) correspond to the tetragonal polymorph of ZrO_2_ and come from planes (011), (110), (112), and (121) according to ICDD card No. 00-050-1089. Within the used temperature range, crystallization of the metastable tetragonal ZrO_2_ is common in thin coatings [46,47,48].

**Figure 8:**
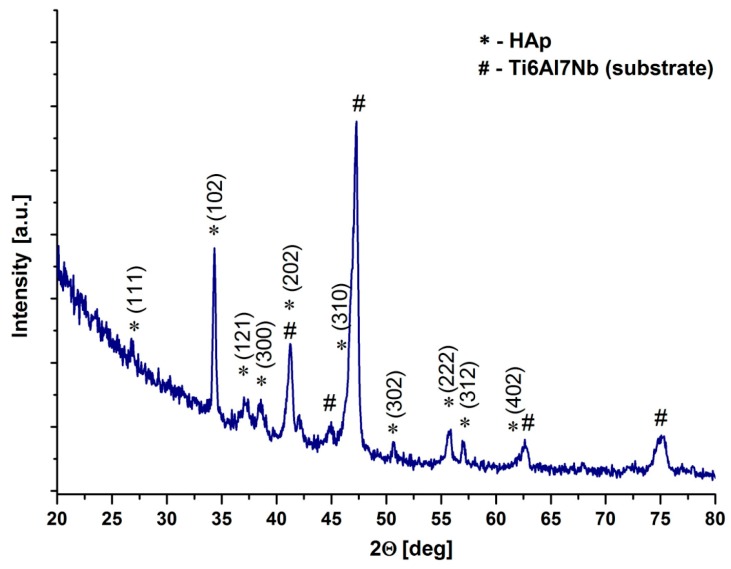
Diffraction pattern of HAp coating deposited on the Ti6Al7Nb alloy substrate.

Figure 8 shows the GIXRD pattern of the HAp coating on the Ti6Al7Nb alloy substrate.

The reflections marked on the diffraction pattern were identified by means of ICDD card No. 01-080-7087 as reflections from the (111), (102), (121), (300), (302), (222), (312), and (402) planes of the hydroxyapatite unit cell provide evidence of the crystalline structure of the coating.

### 3.3. Hardness and Elastic Modulus

The elastic modulus E and the hardness H were determined from the continuously recorded (CSM mode) indentation load displacement curves as proposed by Oliver and Pharr [42].

**Figure 9:**
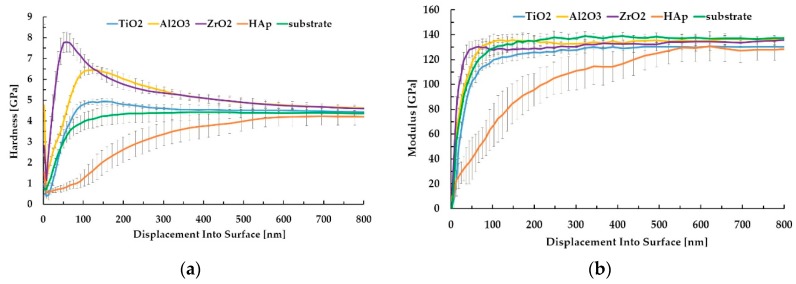
Hardness (**a**) and elastic modulus (**b**) depth profiles for investigated samples.

In Figure 9a, the hardness depth profiles for the investigated one-component coatings are presented compared to the bare substrate.

**Figure 10:**
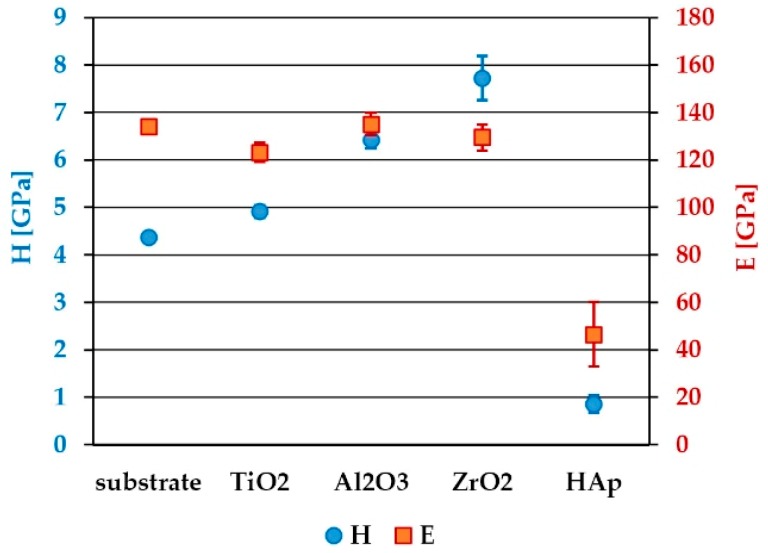
Average values of hardness and elastic modulus for investigated samples.

During the indentation of coated substrates, the maximum penetration depth to which the test can produce substrate independent hardness, and especially the elastic modulus measurements, is assumed at 1/10 of the coating thickness (so called ‘the 10% Rule’). For oxide layers, the hardness was calculated for maximum of the curves at depths ranging approximately from 50 to 120 nm. For the HAp layer, the hardness was calculated at the depth corresponding to curve inflection (approximately 80 nm) from which the influence of the substrate is observed. In Figure 9b, the results of elastic modulus evolution as a function of displacement onto the surface are gathered. It is clearly seen that all the investigated oxide sublayers present a modulus evolution similar to that of the bare substrate, leading to the same range of elastic modulus values. The only exception is the curve evolution for HAp coating where the influence of the substrate on the modulus value extends to the penetration depth of approximately 600 nm. The similarity of the elastic modulus of a coating and substrate can be one of the important factors. A large mismatch between the elastic modulus of the coating and the substrate can cause an increase of tensile stress inside the coating, which can eventually lead to its damage (peeling and cracking) and the cohesive failure of a coating system. The average values of hardness and the elastic modulus for all the measured samples are presented in Figure 10.

The highest value of hardness amongst sol–gel-derived oxide sublayers was obtained for ZrO_2_ (7.73 ± 0.47 GPa), followed by Al_2_O_3_ (6.42 ± 0.19 GPa) and TiO_2_ (4.92 ± 0.14). The Ti6Al7Nb alloy substrate hardness was 4.36 ± 0.07 GPa, while the HAp layer was revealed to be the softest amongst all the samples with hardness not exceeding 1 GPa (0.86 ± 0.18 GPa). In the case of elastic modulus results for all oxide sublayers, the values were similar to that of the bare substrate, ranging from 123 to 135 GPa. For the HAp coating, the obtained value of the elastic modulus was 46.5 ± 13.5 GPa. A relatively high standard deviation for HAp could be explained mainly by the porous and thus nonhomogeneous structure and thickness of the hydroxyapatite layer. The results obtained in our work are mostly in agreement with other papers. According to Fu et al. [49], the nanoindentation hardness of TiO_2_ films may vary within a wide range, from 2 to 18 GPa depending on the crystalline nature and microstructure of the films [50,51]. The nanoindentation results obtained by Borrero-López et al. [50] for TiO_2_ thin films deposited by the filtered cathodic vacuum arc deposition technique on glass confirmed better mechanical properties in the case of crystalline coatings (hardness up to 21 and 18 GPa; modulus up to 221 and 213 GPa for TiO_2_–rutile and TiO_2_–anatase, respectively) compared to amorphous films (hardness 10 GPa, modulus 136 GPa)—higher E and H values ensure a higher load-carrying capacity. Lucca et al. [52] investigated the mechanical properties of zirconia sol–gel coatings deposited on stainless steel prepared by dip coating and subsequently annealed at temperatures in the range of 400–700 °C. The hardness and reduced elastic modulus were measured at depths that meet the 10% rule of the film thickness and were in the range of 2.8 to 6 GPa and 85 to 130 GPa, respectively. Anast et al. [53] measured 600 nm thick sol–gel-derived ZrO_2_ thin films coated on stainless steel fired between coats to temperatures in the range of 500–800 °C, which led to the variation in hardness from 7.1 to 10.3 GPa, depending on the different crystallographic structures obtained.

### 3.4. Scratch Adhesion

**Figure 11:**
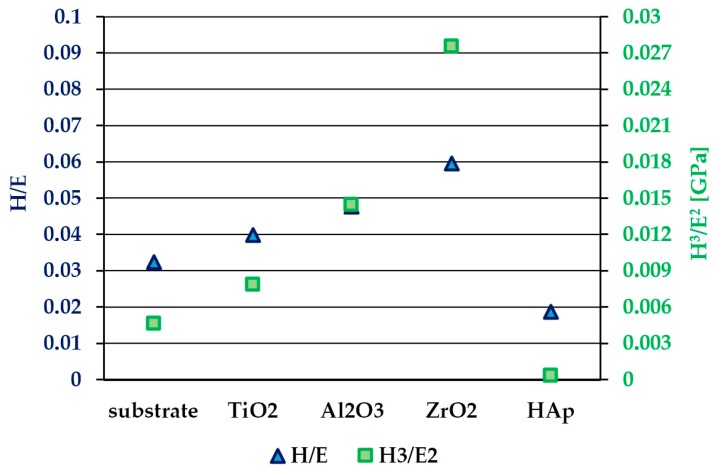
H/E and H^3^/E^2^ ratios results for all the investigated samples.

**Figure 12:**
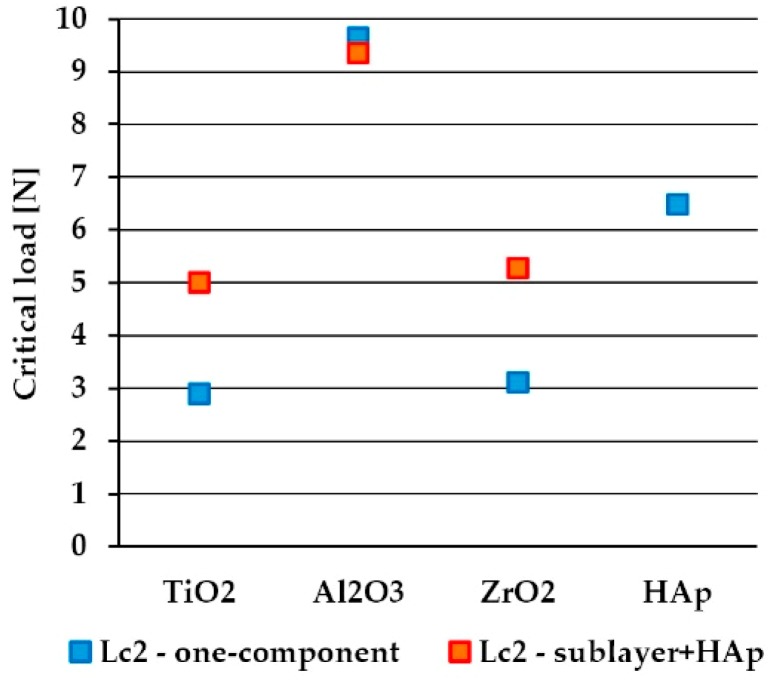
A scratch test critical load Lc2 for one-component and sandwich (sublayer+HAp) composite coatings.

In Figure 11, the results of the H/E and H^3^/E^2^ ratios for all the investigated samples are shown. The results of the scratch test for all the investigated oxides and HAp layers as well as for the sandwich composite coatings (oxide + HAp) are presented in Figure 12. During the scratch tests, increasing the compressive stress leads to coating separation from the substrate either by cracking and lifting (buckling) or by full separation (spallation, chipping). A measure of the adhesion strength of the coating to the substrate is the load at which the coating is fully peeled off from the substrate, which is denoted here as the Lc2 parameter.

The H/E ratio characterizes the resistance of the material to elastic deformation; thus, it can be a better indicator of wear resistance than hardness value only. Leyland and Matthews [54] discussed the significance of elastic strain to failure (which is related to H/E) and fracture toughness in determining tribological behavior. They also pointed out that the H/E ratio is a part of the formula describing the so-called ‘plasticity index’, which is a parameter providing a valuable measure of the elastic regime limit in a surface contact, and it is especially important in wear prediction.

Many factors can influence the adhesion of the coating to the substrate, such as for example the thickness and microstructure of the coating, its surface roughness, and its friction coefficient, as well as resistance to plastic deformation, which is often related to the H^3^/E^2^ parameter [55,56,57]. According to Galvan et al. [57] and Voevodin et al. [58], the increase in the H^3^/E^2^ ratio may lead to both an increase in the elastic recovery and the scratch resistance of TiC-based nanocomposite coatings. Daniel et al. [59] revealed that the scratch adhesion of nc-TiC/a-C:H coatings depends, amongst other parameters, on the H^3^/E^2^ ratio. Beake et al. [60] investigated the relationship between the mechanical properties of thin TiFeN, TiN, and TiFeMoN films and their behavior in nanoscratch tests. For low loads, the response to nanoscratches is governed by the mechanical properties of the coating. Higher scratch resistance is manifested in the cases of the coatings with relatively high H/E parameters and a low plasticity index, while for films exhibiting a very high H/E large area, delamination is observed at higher scratch loads. An optimal combination of mechanical properties (hardness, toughness) providing high resistance to shear can be obtained for the coatings with slightly lower H/E ratios.

The results of scratch tests (Figure 12) on one-component coatings show that the most scratch-resistant coating amongst all the above-mentioned coatings is Al_2_O_3_ (Lc2 = 9.64 N), followed by the HAp coating (Lc2 = 6.48 N). The lowest values of the critical load were achieved for TiO_2_ and ZrO_2_ coatings (2.90 and 3.11 N, respectively). However, according to the results of H/E and H^3^/E^2^, there is no clear evidence for dependence between the scratch adhesion of the synthesized sol–gel coatings and their mechanical properties. One can notice that in case of all three types of oxide coatings, the values of elastic strain to failure (related to H/E) and coating toughness (related to H^3^/E^2^) are mainly governed by the hardness parameter, as for both substrate and oxide coatings, the modulus values are very similar. Unfortunately, there is a lack of research data describing scratch adhesion dependence on the mechanical properties for sol–gel-derived coatings. Kalidindi and Subasri [61] measured adhesion strength by the scratch testing of sol–gel-derived non-fluorinated and fluorinated hydroxyapatite coatings applied on Ti6Al4V titanium alloy. The results revealed the rise of scratch resistance with an increase in annealing temperature (in the range 500–700 °C) from 0.37 to 0.47 N and from 0.38 to 0.57 N in the case of a non-fluorinated and a fluorinated hydroxyapatite, respectively. Rezende et al. [62] reported that from the scratch test curves, the coating failure starts for a load of 25N for a sol–gel-derived Al_2_O_3_ coating on WC tools. Mohseni et al. [23] in the review paper on the adhesion of HAp coating to Ti alloy, summarized the adhesion strength values of HAp coatings on Ti–6Al–4V coated using various techniques. The sputtering technique provides the highest adhesion strength of coating to the substrate (approximately 80 MPa) compared to other methods (amongst others sol–gel methods with an adhesion of approximately 20–28 MPa), which can be attributed to the sputter cleaning and ion bombardment processes. There are also some works referring to application of the interlayer, with H/E being an intermediate value between the substrate and coating, which resulted in improving the wear resistance of the film [63,64,65].

In Figure 12, the results for sandwich composite coatings (oxide sublayer+HAp) are also presented. For both TiO_2_+HAp and ZrO_2_+HAp composite coatings, the Lc2 parameter almost doubled compared to the values achieved for oxide layers only. However, these results are still lower than for the HAp layer deposited directly on the substrate. Surprisingly, in the case of an Al_2_O_3_+HAp sandwich composite coating, the critical load (9.35 N) is almost the same as for the Al_2_O_3_ coating (9.64 N) and much higher than for a one-component HAp coating (6.48 N).

### 3.5. Barrier Properties

**Figure 13:**
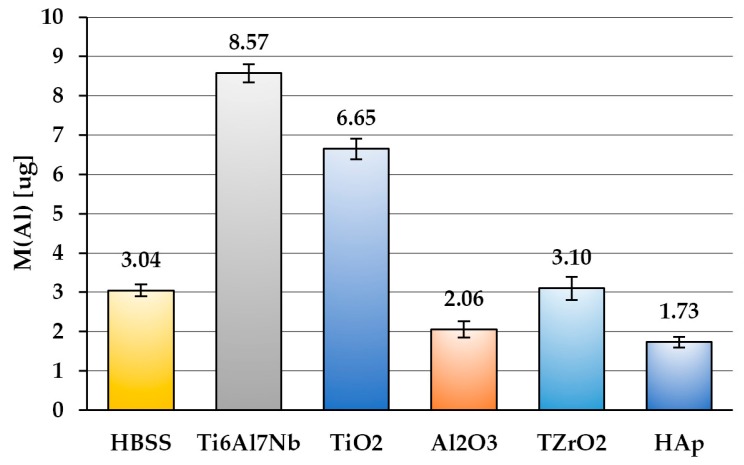
The mass of Al ions released into immersing solutions after a test.

The barrier properties of the one-composite coatings were determined by testing their permeability in an immersion test. It should be noted that the better barrier properties of the coatings, the lower their permeability. The mass of released Al ions found in individual vessels with Hank’s solution after the immersion test, which is calculated according to Formula (1), is presented in Figure 13.

The amount of Al ions in HBSS was the highest for the uncoated Ti6Al7Nb sample. In all the other solutions, a significant decrease of the released ions was recorded compared to the uncoated alloy, which means that all the coatings have reduced the release of Al ions into the environment. The amount of Al ions in HBSS after the test of one-component Al_2_O_3_ and HAp coatings turned out to be less than the initial amount of these ions in HBSS. It follows that these coatings are not only excellent barriers against the permeation of Al ions from the substrate into the environment, but they also have the ability to remove these ions from the solution. The phenomenon of removing metal ions from aqueous solutions by HAp has been extensively studied in the literature. It was found that Hap, owing to its crystal structure and chemical composition, can adsorb metal ions by several mechanisms, including ion exchange, surface complexation, diffusion through the solid material, and dissolution–precipitation [66]. This allows for the purification of aqueous solutions of many metals, for example Cu(II) [67], Pb(II) [68], Sr(II) [69], as well as Cr(III) [70] and Al(III) [66]. The ability of Al_2_O_3_ coatings for removing Al ions from immersion solution may result from its amorphous structure and possible non-stoichiometric chemical composition.

The permeability of coatings for Al ions, calculated according to Formula (2) as the mass of Al ions released into soaking solution per surface unit, is presented in Table 4.

It was found that the mass of Al ions released from the bare Ti6Al7Nb substrate into the immersion solution is similar to that found in other studies [71]. One can notice that all tested coatings restrained the permeation of Al ions into the environment but significantly differ in barrier properties. TiO_2_ turned out to be the least effective barrier coating, while the ZrO_2_ coating reduced the permeability of Al ions almost 100 times compared to an uncoated substrate, whereas Al_2_O_3_ and HAp completely blocked their permeation. However, in the case of Hap, the barrier effect may not be permanent due to the possibility of the dissolution of this layer.

## 4. Summary and Conclusions

The titania (TiO_2_), alumina (Al_2_O_3_), zirconia (ZrO_2_), and hydroxyapatite (HAp) coatings as well as sandwich composite (oxide+HAp) coatings of good quality were produced by the sol–gel method.

All the prepared coatings, except for Al_2_O_3_, revealed a crystalline structure. Obtained oxide layers were characterized by relatively small roughness parameters as compared to the HAp layer (an approximately 10-fold increase of roughness was observed). The thickness of the HAp layer was at least twice as high as that compared to the oxide sublayer. The obtained parameters of thickness and roughness benefit both the oxide sublayers and the HAp outer layer.

As a result of hardness, modulus of elasticity, and adhesion tests of individual layers as well as composite oxide+HAp coatings, it was found that:

- elastic strain to failure and the toughness of the investigated oxide layers are mainly governed by the hardness parameter;
- no relation between the mechanical properties and scratch resistance of individual layers has been found;
- the best scratch resistance was observed for sandwich composite coatings with Al_2_O_3_ as a sublayer (Al_2_O_3_+HAp).

In the absence of a relationship between mechanical properties and adhesion, it can be assumed that factors other than mechanical properties may have a greater effect on adhesion. One such factor may be the chemical structure of the coating, which allows it to chemically bond to the substrate. It can be assumed that both the formation process and the resulting amorphous structure of the obtained alumina layer can promote such chemical connections with the metallic substrate. An improvement in the adhesion of sandwich composite coatings compared to HAp deposited directly onto the substrate was only noted for the Al_2_O_3_ sublayer. Other studies [10] have reported that also TiO_2_ and ZrO_2_ sublayers show a visible improvement in HAp adhesion. Such a difference in the obtained results indicates a significant influence of the coatings preparation method on their properties.

The examination of barrier properties showed significant differences in the behavior of individual coatings. All of the analyzed coatings limited the permeation of Al ions from the Ti6Al7Nb substrate, although to varying degrees. The Al_2_O_3_ and HAp coatings showed the best barrier properties. ZrO_2_ also was a very good barrier for Al ions. The least barrier properties revealed TiO_2_. Thus, in oxide+HAp composites, the best barriers were the Al_2_O_3_ and ZrO_2_ sublayers, because they have limited the permeation of Al ions into the tissue environment the most effectively, regardless of the presence of the HAp layer.

The obtained results showed that the Al_2_O_3_ sublayer revealed the best properties compared to the other tested sublayers. The Al_2_O_3_ sublayer improved the adhesion of the sandwich composite Al_2_O_3_+HAp coating and at the same time had excellent barrier properties.

## Figures and Tables

**Table 1:** Parameters of coating processes.

Coating Type	Pulling Out Speed [mm/s]	Drying Time [Minutes]	Heat Treatment Temperature [°C]	Heat Treatment Time [Minutes]
TiO_2_	1.2	15	500	15
Al_2_O_3_				
ZrO_2_				
HAp	1.2	−	500	20

**Table 2:** Ra and Rz parameters of Ti6Al7Nb substrate and deposited one-component coatings.

	Ti6Al7Nb	TiO_2_	Al_2_O_3_	ZrO_2_	HAp
Ra [nm]	0.34 ± 0.10	2.27 ± 0.34	0.60 ± 0.27	1.45 ± 1.34	29.1 ± 7.82
Rz [nm]	2.37 ± 0.66	15.3 ± 2.96	2.85 ± 1.47	8.69 ± 7.74	131 ± 23.2

**Table 3:** Thickness of one-component oxide and hydroxyapatite (HAp) coatings.

	TiO_2_	Al_2_O_3_	ZrO_2_	TZrO_2_	HAp
Thickness [nm]	81.49 ± 0.30	86.48 ± 0.46	70.93 ± 0.62	48.8 ± 0.11	175 ± 25

**Table 4:** Permeation of Al ions for all one-component coatings and uncovered Ti6Al7Nb substrate.

	Ti6Al7Nb	TiO_2_	Al_2_O_3_	TZrO_2_	HAp
Permeation of Al ions [µg/cm^2^]	1.072 ± 0.044	0.708 ± 0.052	0	0.011 ± 0.023	0
